# A novel family of mammalian transmembrane proteins involved in cholesterol transport

**DOI:** 10.1038/s41598-017-07077-z

**Published:** 2017-08-07

**Authors:** Kevin M. Méndez-Acevedo, Victor Julián Valdes, Alexander Asanov, Luis Vaca

**Affiliations:** 1 Departamento de Biología Celular y del Desarrollo, Instituto de Fisiología Celular, Universidad Nacional Autónoma de Mexico, Mexico city, Mexico; 2 Icahn School of Medicine at Mount Sinai, Black Family Stem Cell Institute, New York, NY USA; 3 TIRF Labs Inc. 106 Grendon Place, Cary, NC 27519 USA

## Abstract

Cholesterol is an essential compound in mammalian cells because it is involved in a wide range of functions, including as a key component of membranes, precursor of important molecules such as hormones, bile acids and vitamin D. The cholesterol transport across the circulatory system is a well-known process in contrast to the intracellular cholesterol transport, which is poorly understood. Recently in our laboratory, we identified a novel protein in *C. elegans* involved in dietary cholesterol uptake, which we have named ChUP-1. Insillicoanalysis identified two putative orthologue candidate proteins in mammals. The proteins SIDT1 and SIDT2 share identity and conserved cholesterol binding (CRAC) domains with *C. elegans* ChUP-1. Both mammalian proteins are annotated as RNA transporters in databases. In the present study, we show evidence indicating that SIDT1 and SIDT2 not only do not transport RNA, but they are involved in cholesterol transport. Furthermore, we show that single point mutations directed to disrupt the CRAC domains of both proteins prevent FRET between SIDT1 and SIDT2 and the cholesterol analogue dehydroergosterol (DHE) and alter cholesterol transport.

## Introduction

Cholesterol is an essential molecule in mammals not only for its structural function in cell membranes, where it regulates stability, fluidity, integrity and permeability^1, 2^. Cholesterol also plays a significant role as a signaling molecule in the cells and is a precursor of other important molecules such as steroid hormones, bile acids and vitamin D.

Due to the properties of cholesterol, as a highly hydrophobic molecule, it needs specialized transport mechanisms such as in the circulatory system, where it is carried as a component of lipoproteins^3^. This mechanism of cholesterol transport is well known, unlike the intracellular cholesterol transport, which remains poorly understood^4–9^. Several proteins have been identified to interact directly with cholesterol^10^. The ABC transporters are one of the most studied proteins involved in specific sterol transport across the plasma membrane of cells^6–8^. Another example of specific uptake is the transport protein NPC1L1, which is directly involved in the uptake of free cholesterol from the luminal space of the intestine and into enterocytes^11–15^. Once inside the cell, the mechanisms of cholesterol transport and redistribution and the proteins involved in such tasks remain largely unidentified^8^. There have been proposed three different mechanisms to regulate the cholesterol dynamics inside the cell^5^: transport diffusion, through contact sites between adjacent membranes and cytoplasm transport using carrier proteins.

Several proteins and protein domains have been identified to interact with cholesterol^10, 16–28^. One of such domains in proteins is the so-called cholesterol recognition/interaction amino acid Consensus (CRAC) domain^29, 30^. The sequence of this motif is characterized by the presence of the following amino acids: V/L-X(1-5)-Y-X(1-5)-R/K. The tyrosine is particularly relevant because of its interaction with the OH-polar group in the cholesterol molecule. This motif is present in a broad range of proteins involved in different functions in transport, metabolism and regulation of cholesterol^30^.

Recently our group identified a novel protein involved in cholesterol transport in the nematode *Caenorhabditis elegans* ^31^. Because *C. elegans* is auxotroph for cholesterol, it depends entirely on the diet to supplement this important molecule. ChUP-1 is a 9-transmembrane domain protein involved in dietary cholesterol uptake, composed of 2 CRAC domains. We have previously reported that *in silico* analysis identified two putative mammalian orthologues (SIDT1 and SIDT2). Both mammalian proteins are annotated as homologues of the *C. elegans* RNA transporter SID-1. However, we have previously shown that the greater homology lies between these two proteins and ChUP-1, not only at the amino acid level but also they have two putative CRAC domains located in similar regions to ChUP-1 and all form proteins with putative 9-transmembrane domains.

In the present study, we have cloned human SIDT1 and SIDT2 and produced fusion proteins to the green fluorescent protein (GFP). Using a wide variety of methods, we show evidence strongly suggesting that SIDT1 and SIDT2 are involved in cellular cholesterol transport in mammalian cells. Site-directed mutagenesis directed to disrupt putative CRAC domains indicate that the domain located in the transmembrane region of both proteins is involved in cholesterol binding. Disruption of this CRAC domain prevents FRET between SIDT1 and SIDT2 proteins and the cholesterol analogue dehydroergosterol (DHE) while affecting the uptake of [^3^H]-Cholesterol. Most interestingly, removal of cholesterol from the plasma membrane (PM) induces the translocation of SIDT1 from intracellular compartments to the PM. This translocation requires clathrin, as reducing clathrin levels with RNA interference (RNAi) results in diminished SIDT1 translocation to the PM and prevents the uptake of [^3^H]-Cholesterol. While SIDT1 participates in the uptake of cholesterol from the extracellular medium and into the cell, the localization of SIDT2 is in intracellular compartments, most likely the endoplasmic reticulum and Golgi apparatus. SIDT2 associates to intracellular cholesterol as evidenced by FRET experiments and may be involved in the redistribution of cholesterol among organelles. Interestingly, SIDT2 does not participate in cholesterol uptake from the extracellular space, as evidenced by the lack of [^3^H]-Cholesterol uptake.

All the evidence gathered in the present study indicates that SIDT1 and SIDT2 are members of a novel family of proteins involved in the uptake and intracellular cholesterol transport in mammals.

## Results

### SIDT1 and SDIT2 are involved in cholesterol but not dsRNA transport

**Figure 1:**
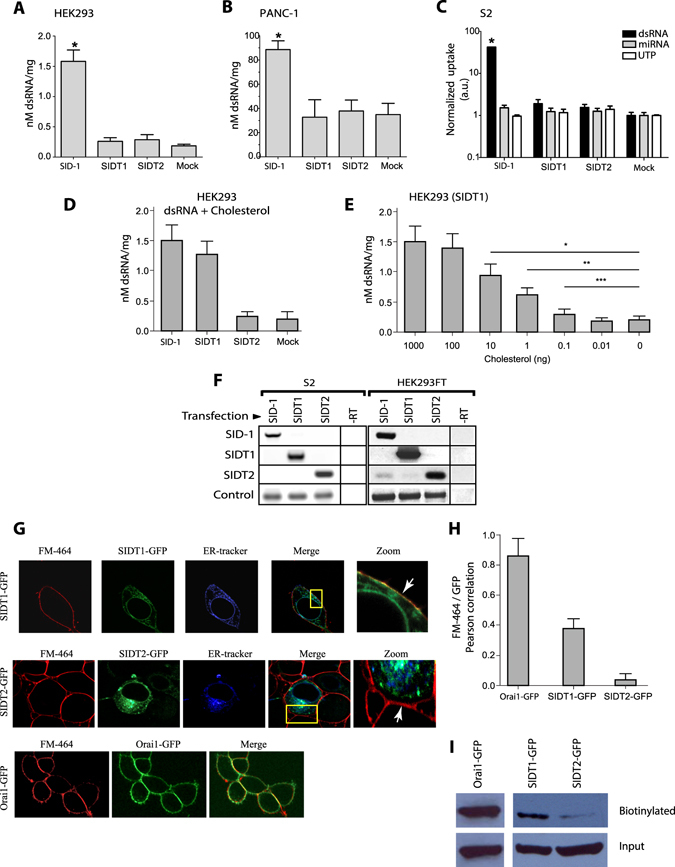
Overexpression of SIDT1 or SIDT2 does not increase dsRNA uptake. HEK293 FT cells (**A**), PANC-1 cells (**B**) and S2 cells were transfected with SID-1 (from *C. elegans*), SDIT1 (human), SDIT2 (mouse) or empty GFP vector (Mock) and incubated with 5 ug/ml of radioactive dsRNA (700 nucleotides) for 2 hours at 37 °C. The molarity was calculated with the sequence of dsRNA and then normalized with the total protein of each sample. Error bar: SD; n > 5; *p < 0.01. (**C**) S2 cells from *Drosophila melanogaster* incubated for 4 hours at 37 °C with dsRNA (700 nt length), miRNA (pre-let-a) or the equivalent in counts per minute (c.p.m)of 32P-γ-UTP. The uptake was normalized with the total protein for each sample; a.u. arbitrary units. (**D**) Mixing 5 µg/ml of radioactive dsRNA with 10 ng of cholesterol results in dsRNA uptake in HEK293 cells overexpressing SIDT1, SIDT2, SID-1 or the empty vector. Values show the mean ± standard deviation of the amount of dsRNA incorporated per mg of total protein in the cell lysates. (**E**) Dose-dependent uptake of radioactive dsRNA-dependent on the cholesterol content in the mix (0 to 1000 ng of cholesterol). *p < 0.001; **p < 0.01; ***p < 0.05. (**F**) RT-PCR of each transfection in S2 cells or HEK293 FT cells. The actin gene (S2) and GAPDH (HEK293) were used as controls. -RT control without reverse transcriptase. (**G**) Confocal microscopy colocalization studies for SDIT1 or SIDT2 using FM-464 as plasma membrane marker and ER-tracker for the endoplasmic reticulum (ER). Notice in the zoom that SIDT1 shows very high co-localization with FM-464 whereas SIDT2 shows an intracellular localization co-localizing with ER tracker. (**H**) Mean ± standard deviation for Pearson’s correlation coefficient between FM-464 and GFP. Notice the very high co-localization between Orai1 channel, which was used as plasma membrane (PM) localization control with FM-464. SIDT1 shows very high co-localization as well, compared with the poor co-localization of SIDT2. (**I**) Biotinylation studies for Orai1-GFP, SIDT1-GFP and SIDT2-GFP. Notice the subtle amount of SDIT2-GFP found at the plasma membrane when compared to SIDT1-GFP and Orai1-GFP. All conditions were included in a single figure and no other bands were detected.

Previous studies have identified SIDT1 and SIDT2 as homologues of the *C. elegans* SID1 protein, which is involved in the transport of double-stranded RNA (dsRNA) in the nematode^32, 33^. One of such studies showed the transport of dsRNA in PANC-1 cells overexpressing SIDT1. Databases have both genes annotated as dsRNA transporters (NCBI, SIDT1 Gene ID:54847 and SIDT2 Gene ID: 51092). We have shown previously that both SIDT1 and SIDT2 shared a higher identity with ChUP-1 than with SID1 (31) (Supplementary Fig. 1). We have reported previously that ChUP-1 is a novel protein involved in dietary cholesterol uptake in the nematode *C. elegans*. We conducted experiments with radioactive dsRNA using *C. elegans* SID1 as positive control. Transport experiments show that neither SIDT1 nor SIDT2 transport dsRNA or microRNAs in HEK293, PANC-1 or S2 *Drosophila melanogaster* cells (Fig. 1A–C). Most interestingly, mixing dsRNA with cholesterol resulted in increased uptake of dsRNA (Fig. 1D). The transport of the dsRNA was dose-dependent on the amount of cholesterol used (Fig. 1E). The expression of SID-1, SIDT1and SIDT2 was corroborated with RT-PCR (Fig. 1F).

The fusion protein SIDT1-GFP shows high co-localization with ER-tracker (a marker for the endoplasmic reticulum) and FM-464, a marker of the plasma membrane, while SIDT2 shows co-localization almost exclusively with ER-tracker (Fig. 1G). The co-localization between SIDT2 and FM-464 was insignificant (Fig. 1H). As positive control, we used the channel Orai1, which is present only at the plasma membrane^34^. Biotinylation experiments confirmed that SIDT1 is present at the plasma membrane while SIDT2 is not (Fig. 1I).

**Figure 2:**
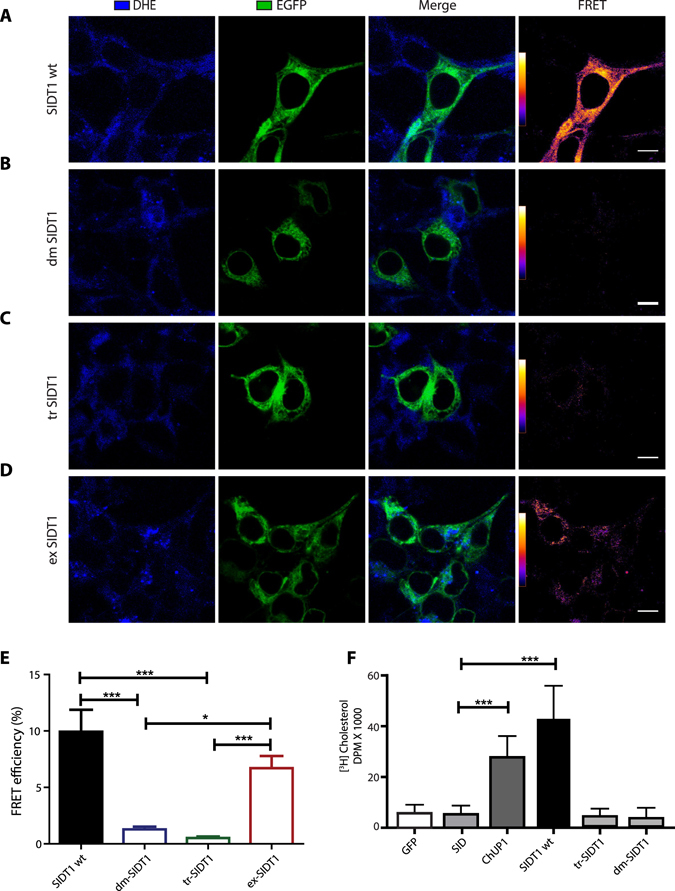
The localization of SIDT1 and cholesterol transport. (**A**) Confocal microscopy representative images illustrating the localization of the SIDT1 wild-type, (**B**) double CRAC domain mutant (dmSIDT1), (**C**) transmembrane CRAC domain (tmSIDT1) and (**D**) extracellular CRAC domain (exSIDT1). All proteins were fused to GFP. In blue is illustrated the fluorescence of dehydroergosterol (DHE). FRET signal was calculated using the sensitized emission method^44, 45^. Scale bar: 10 microns. (**E**) FRET efficiency between GFP and DHE (plotted as percentage). (**F**) [^3^H]-cholesterol uptake from HEK293 cells expressing the SIDT1 wild type and the three mutants. *p < 0.001; **p < 0.01; ***p < 0.05.

To further explore the association of SIDT1 and SIDT2 to cholesterol, we conducted FRET experiments using the cholesterol fluorescent analogue dehydroergosterol (DHE)^31^. Since both proteins carry putative CRAC domains, we produced mutants with the CRAC domains disrupted. Mutations in the transmembrane CRAC domain prevented FRET between SIDT1 and DHE (Fig. 2). The extracellular CRAC domain does not appear to be involved in DHE association (Fig. 2A–F). Disruption of the transmembrane CRAC domain or the double mutant not only prevented FRET between SIDT1 and DHE but also prevented [^3^H]-Cholesterol uptake (Fig. 2F). Even though SIDT2 is not involved in [^3^H]-Cholesterol uptake from the extracellular space, we found that intracellularly located SIDT2 generates a robust FRET signal with DHE, which can be prevented when using the mutant with the transmembrane CRAC domain disrupted or the double mutant (Supplementary Fig. 2). Furthermore, SIDT2 shows strong co-localization with golgin-97, a protein resident of the trans-Golgi network (TGN)^35^, (Supplementary Fig. 3). All these results strongly suggest that SIDT2 is involved in the intracellular transport of cholesterol.

### Reducing cholesterol from the plasma membrane triggers the translocation of SIDT1 to the PM

**Figure 3:**
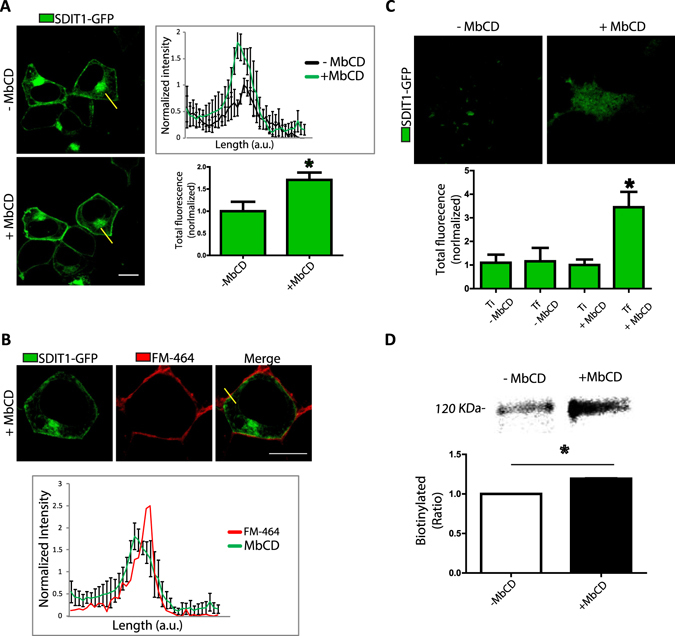
Cholesterol reduction induces the relocalization of SIDT1 to the plasma membrane. (**A**) SIDT1-GFP localization observed by confocal microscopy in HEK293 cells. The yellow line indicates the line where fluorescence was measured to detect the intensity levels of SIDT1-GFP before and after methyl beta cyclodextrin (MβCD) treatment for 40 minutes. The right panel shows the fluorescence intensity along the line before and after MβCD. Data shows mean ± standard deviation from at least 40 independent cells. Length plotted in arbitrary units (a.u.). Lower right panel shows the mean ± standard deviation as normalized fluorescence (1 is the before MβCD condition). Scale bar: 10 microns. (**B**) Co-localization of SIDT1-GFP fluorescence signal with the plasma membrane marker FM-464. (**C**) Total internal reflection fluorescence microscopy (TIRFM) representative images of the localization of SIDT1-GFP before and after MβCD treatment. The lower panel shows the mean ± standard deviation of normalized fluorescence intensity obtained from at least 40 independent experiments. Data shows initial recording time (Ti) and final recording time (Tf). Notice that Ti and Tf do not change in cells not exposed to MβCD. (**D**) Biotinylation analysis of SIDT1-GFP before and after MβCD treatment. The lower panel shows the mean ± standard deviation of biotinylated protein obtained from measuring the density of the protein bands in the gel from at least 4 independent experiments. Gels were cropped to include all conditions in a single figure and because no other bands were detected in the rest of the gel.

Since SIDT1 is present mainly at the plasma membrane (PM) and involved in the uptake of cholesterol, we explored the effect of reducing PM cholesterol using the agent methyl beta cyclodextrin (MβCD). Interestingly, treating the cells for 40 minutes with MβCD resulted in increased SIDT1 at the plasma membrane (Fig. 3A). The increment of SIDT1 was evident using line analysis of the regions in cells labeled with the plasma membrane marker, FM-464 (Fig. 3A). Using total internal reflection fluorescence microscopy (TIRFM) showed increased SIDT1-GFP fluorescence near the plasma membrane after MβCD treatment (Fig. 3C). This observation was confirmed by biotinylation studies (Fig. 3D).

**Figure 4:**
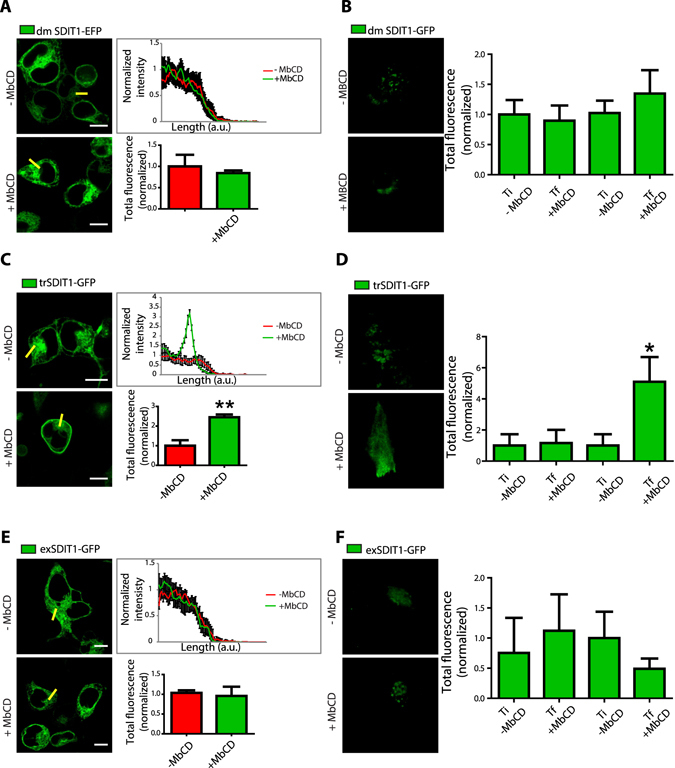
The extracellular CRAC domain from SIDT1 is part of a vesiculation signal required for translocation to the plasma membrane. (**A**) Representative confocal images of HEK293 cells expressing the SIDT1 double mutant from the CRAC domains (dmSIDT1). The right panel shows the line analysis illustrating the lack of translocation of the dmSIDT1 upon MβCD treatment. The lower panel shows the mean ± standard deviation of the fluorescence intensity in the line before and after MβCD treatment obtained from at least 40 independent experiments. (**B**) Total internal reflection fluorescence microscopy (TIRFM) representative images obtained with HEK293 cells expressing the dmSIDT1. The right panel shows the mean ± standard deviation from the total fluorescence of at least 30 independent measurements. (**C**) Representative confocal images of HEK293 cells expressing the SIDT1 transmembrane mutant (tmSIDT1). Notice the normal translocation to the plasma membrane after MβCD treatment. (**D**) Total internal reflection fluorescence microscopy (TIRFM) representative images obtained with the trSIDT1 mutant. (**E**) Representative confocal images of HEK293 cells expressing the exSIDT1 mutant. Notice that this mutant does not translocate to the plasma membrane after MβCD treatment. (**F**) Total internal reflection fluorescence microscopy (TIRFM) representative images obtained with the exSIDT1 mutant. Ti, the initial time of recording and Tf, final recording time. Total fluorescence normalized to Ti. In all panels scale bar: 10 microns.

To study further the translocation of SIDT1 to the PM, we conducted experiments with the mutants from the transmembrane and extracellular CRAC domains and the double mutant. Most interestingly, disruption of the transmembrane CRAC domain did not alter the translocation (Fig. 4D,E), but the disruption of the extracellular CRAC domain prevented translocation of SIDT1 upon removal of cholesterol with MβCD (Fig. 4C,D). Similar results were obtained with the double mutant. The extracellular CRAC domain overlaps with a vesiculation signal (Yxxɸ), which is recognized by specific accessory proteins to form vesicles like AP-2 or Dab-2^36^. Disruption of the vesiculation signal prevents the translocation of SIDT1 to the PM. To study this transport process in greater detail, we explored several proteins involved in vesicle transport. Because the vesiculation signal (Yxxɸ) was originally identified in cargo complexes found in clathrin-based vesiculation from the plasma membrane and the trans-Golgi network^37^, we decided to explore the role of clathrin in the translocation of SIDT1.

**Figure 5:**
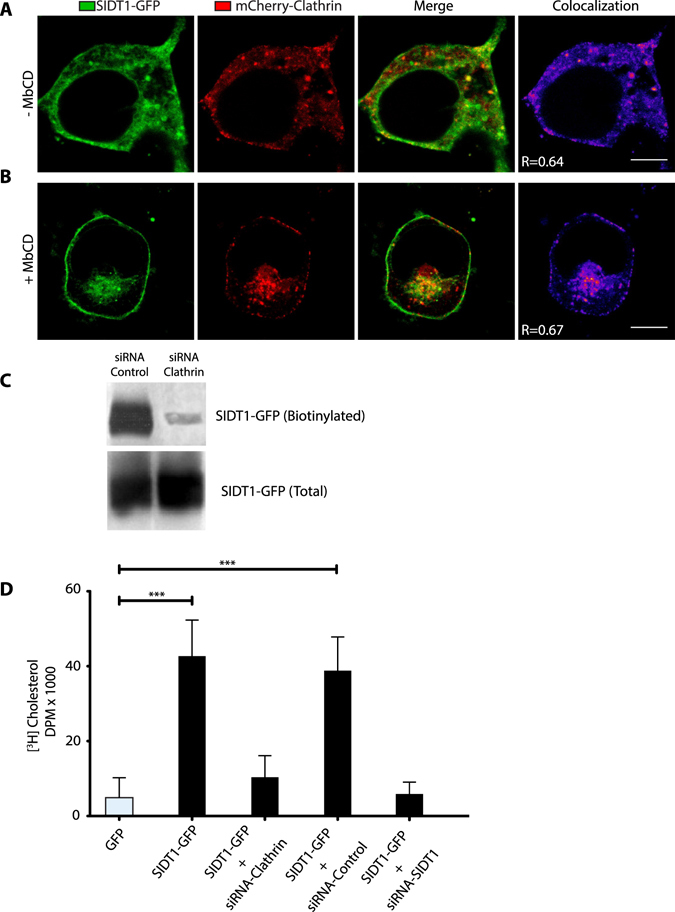
The re-localization of SDIT1 depends on clathrin. Confocal microscopy images illustrating the localization of SIDT1-GFP (green), mCherry-Clathrin (red) and the co-localization panel before (**A**) and after (**B**) MβCD treatment. (**C**) Biotinylation studies illustrating that siRNA against clathrin reduces the amount of SIDT1-GFP biotinylated. Gels were cropped to include all conditions in a single figure and because no other bands were detected in the rest of the gel. (**D**) [^3^H]-cholesterol uptake in cells treated with a siRNA for clathrin, control (scramble siRNA) or siRNA for SIDT1. ***p < 0.001. Scale bar: 10 microns.

Clathrin showed strong colocalization with SIDT1 (Fig. 5A,B). Most notably, RNAi against clathrin prevented the translocation of SIDT1 to the PMafter MβCD treatment (Fig. 5C) and eliminated[^3^H]-Cholesterol uptake (Fig. 5D).

### Selective sterol binding from the CRAC domain in SIDT1

**Figure 6:**
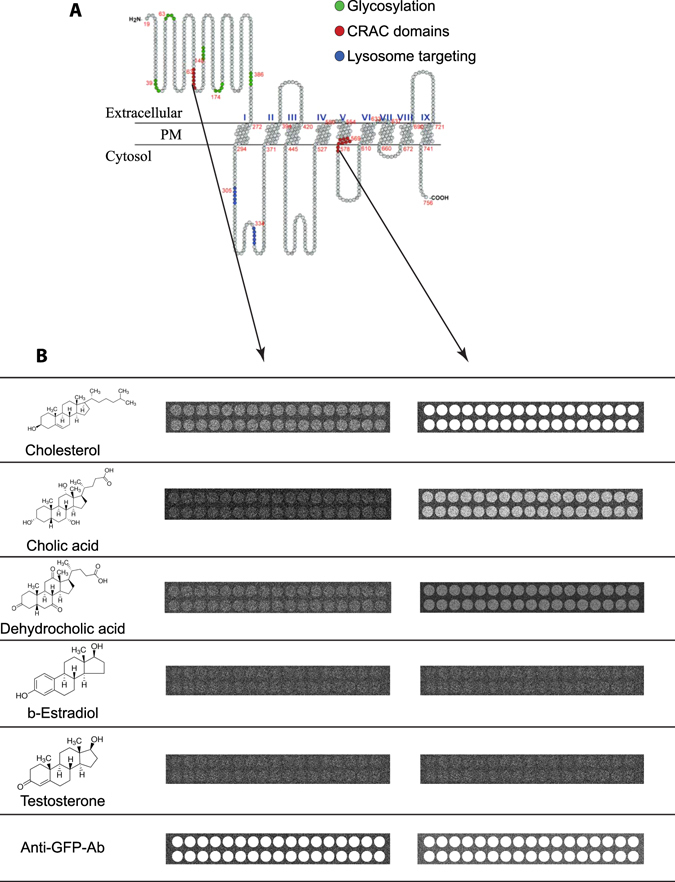
Sterol microarrays suggest that the transmembrane CRAC domain from SIDT1 binds several sterols. (**A**) Cartoon illustrating the putative secondary structure of SIDT1. The nine putative transmembrane domains are shown. In red are mark the amino acids forming the two CRAC domains and in blue a putative lysosome localization signal (not explored in the present study). (**B**) TIRFM-based microarray studies illustrating the spots containing the following sterols tested: cholesterol, cholic acid, dehydrocholic acid, β-estradiol and testosterone. As positive control, some spots in the microarray were printed with an anti-GFP antibody (lower panels). Each microarray contained 2 lines of 17 spots each. The fluorescence illustrated represents the binding of the fusion protein formed by the CRAC domain attached to GFP. For CRAC domain sequences, please refer to Material and Methods. Notice that the extracellular CRAC domain did not bind any of the sterols tested while the transmembrane CRAC domain binds to cholesterol and only weakly to cholic acid. Both fusion proteins bind strongly to the anti-GFP antibody.

To study the binding of different sterols to the CRAC domains from SIDT1 we produced fusion proteins between the CRAC domains and GFP. We developed a novel high-throughput screening system based on TIRFM and microarrays of different sterols spotted on the glass. We measured the amount of the CRAC-GFP peptide bound to the TIRFM microarray using the fluorescence of GFP as a reporter. Most interestingly, the extracellular CRAC domain did not bind any of the sterols tested (Fig. 6A,B). As a positive binding control, we utilized an anti-GFP antibody spotted on the microarrays. In contrast, the transmembrane CRAC domain bound cholesterol and cholic acid, only weakly dehydrocholic acid but noβ-estradiol or testosterone (Fig. 6B). These results in combination with the [^3^H]-Cholesterol uptake and FRET interactions strongly suggest that the transmembrane CRAC domain from SIDT1 is responsible for binding cholesterol (and perhaps other sterols) and the extracellular CRAC domain is not functional but forms part of a signal required for the translocation of SIDT1 to the PM upon reduction of cholesterol at the plasma membrane.

## Discussion

The *in silico* analysis previously performed by our group identified putative mammalian homologues to *C. elegans* ChUP-1 protein, which is involved in cholesterol uptake in the digestive tract of the nematode^31^. Mammalian SIDT1 (Gene ID: 54847) and SIDT2 (Gene ID: 51092) are annotated in National Center for Biotechnology Information (NCBI) as homologues of the dsRNA transporter SID1 from *C. elegans*. However, we showed previously that the highest identity is with ChUP-1^31^. Even though a couple of studies have shown dsRNA transport by SIDT1^32, 33^. A recent study suggested that dsRNA transport by SIDT1 was attained only when the dsRNA was covalently bound to cholesterol^38^. Furthermore, this study shows that reduction of SIDT1 protein via knockdown with RNAi prevented the transport of dsRNA covalently attached to cholesterol^38^. In the present study, we show that dsRNA transport can be attained when mixed with cholesterol (Fig. 1). These evidences strongly suggest that SIDT1 may transport dsRNA associated with cholesterol, but the primary role of SIDT1 is the transport of sterols. Our FRET studies demonstrate the direct interaction between SDIT1 and cholesterol, mediated by a CRAC domain in the protein. This interaction was disrupted by the mutagenesis of the central tyrosine of the domain^26, 27^. The SIDT1-mediated uptake of [^3^H]-Cholesterol further strengthens this hypothesis. [^3^H]-Cholesterol uptake is abolished when the central tyrosine of the domain was replaced by glycine.

**Figure 7:**
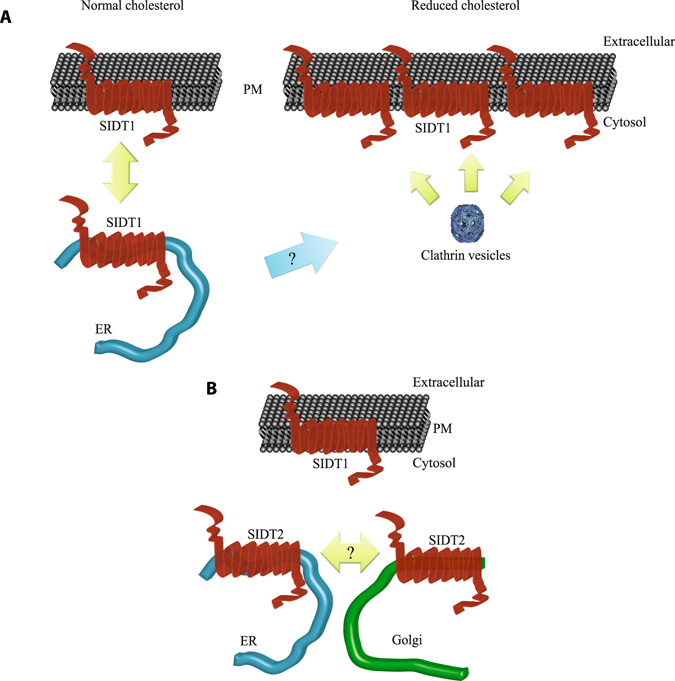
Cartoon illustrating the model proposed for cholesterol transport mediated by SIDT1 and SIDT2. (**A**) Under normal cholesterol plasma membrane levels, SIDT1 is present in the plasma membrane and endoplasmic reticulum. Upon reduction of plasma membrane cholesterol with MβCD, SIDT1 moves to the plasma membrane by a clathrin-dependent mechanism. This newly incorporated SIDT1 may come from the endoplasmic reticulum or vesicles located near the plasma membrane. (**B**) While SIDT1 is present in the plasma membrane, SIDT2 is found primarily in the endoplasmic reticulum and the Golgi apparatus. SIDT2 may be involved in the transport of cholesterol between these two organelles.

Due to its cellular localization, SIDT1 (which is predominantly found in plasma membrane) appears to carry a role in the uptake of cholesterol from the extracellular space; such function is performed via binding of cholesterol to the transmembrane CRAC domain of the protein. This suggests that cholesterol may be incorporated into the plasma membrane by diffusion and SIDT1 binds the cholesterol to incorporate it into the cell interior via endocytosis of the SIDT1-cholesterol complex. SIDT2, on the other hand, co-localizes with the endoplasmic reticulum and Golgi apparatus. This intracellular localization suggests that SIDT2 may participate in the re-localization of cholesterol among organelles (Fig. 7). This observation is confirmed by the robust FRET signal between SIDT2 and DHE. Both proteins (SIDT1 and SIDT2) bind cholesterol via their transmembrane CRAC domains, as evidenced by single point mutations directed to disrupt these domains. The extracellular CRAC domains in SIDT1 and SIDT2 overlap with a putative vesiculation signal (Yxxɸ). This vesiculation signal was originally identified in cargo complexes found in clathrin-based vesiculation from the plasma membrane and the trans-Golgi network^37^.

Most interestingly, disruption of this vesiculation signal prevents the translocation of SIDT1 to the plasma membrane upon removal of cholesterol from the PM. Similar results are obtained if RNAi is directed to knock down clathrin. Clathrin knockdown prevents SIDT1 mediated [^3^H]-cholesterol uptake (Fig. 5). The translocation of SIDT1 upon reduction of cholesterol is not unique, other proteins involved in cholesterol transport like the sterol regulatory element-binding protein (SREBP), which acts as a lipid sensor in the endoplasmic reticulum to maintain the homeostasis of lipids in the cell, also shows translocation^6, 8, 10, 23, 39^. This vesiculation signal (Yxxɸ) is found in SIDT2 as well, but its functional significance was not evaluated in the present study. Measuring the translocation of cholesterol between organelles in living cells is a fairly complex task.

Both SIDT1 and SIDT2 are present in a wide variety of tissues in mammals. The expression of SDIT1 seems to be more important in the late developmental stages of mice, and it continues to be highly expressed in the adult (Supplementary Fig. 4). In contrast, SDIT2 is mainly expressed in early developmental stages of the mouse (Supplementary Fig. 4). Our data suggests that SDIT1 is primarily expressed in the brain and thymus (Supplementary Fig. 4).

Cholesterol plays a key role in nerve regeneration in the adult brain^40^. Furthermore, reduction in SREBP protein alters oligodendrocyte differentiation^41^ and may play a role in schizophrenia^42^. The role of SIDT1 in the uptake of cholesterol in the CNS is a subject of future investigations.

## Materials and Methods

### Reagents

All reagents used in this study were of analytical grade from Sigma (Saint Louis, MO) unless otherwise stated. Membrane marker FM4-64 was purchased from Invitrogene (Carlsbad, CA).

### Plasmids

SID-1 was cloned from *c elegans* as previously described^31^. SIDT1 was cloned from the human cDNA clone HFLJ from Riken Consortium (Japan) using the following primers: TCAGATCTGCACCGGGCTTTGGAA and GTGGATCCCAGAAGACAGGGATCTGGTCTC. The product was cloned in pEGFP-N1 from Clontech (Mountain View, CA. USA). Details of the cloning can be found in ref. 42. The mcherry-clathrin plasmid was purchased from AddGene. pRMHA3 plasmid was used to clone each protein for the overexpression in S2 (Drosophila melanogaster) cells, EndoCyan (a Rho GTPase localized in endosomes).

### Uptake of dsRNA in cells

dsRNA was obtained using a T7 polymerase(NEBL) and α32P UTP (Perkin Elmer) from a generic primer for T7^43^. Cells overexpressing each one of the proteins were incubated in Optimem with 5 µg/ml of dsRNA (700 nucleotides of length) at 37 °C for 2 hours^43^. Then the cells were washed with PBS and lisated, the radioactivity was measured using a scintillation counter and was normalized against the total amount of protein.

### Site-directed mutagenesis of CRAC domains

SIDT1-GFP and SIDT2-GFP CRAC single amino acid changes were generated using QuikChange® Site-Directed Mutagenesis Kit (Stratagene, Santa Clara, CA. USA) according to manufacturer’s instructions using the following primers.

SIDT1 transmembrane CRAC domain:

Forward 5′- GCCCTCAGCACCCAGATAGGTTATATGGGTCGTTTCAAG -3′

Reverse 5′- CTTGAAACGACCCATATAACCTATCTGGGTGCTGAGGGC -3′

SIDT1 extracellular CRAC domain:

Forward 5′- CCCCTGGGTGCTCAGGGCAAACTGCTAGTTAC -3′

Reverse 5′- GTAACTAGCAGTTTGCCCTGAGCACCCAGGGG -3′

SIDT2 transmembrane CRAC domain:

forward 5′- CTCAGCACTCAGCTCGGTTACATGGGCCGCTG -3′

reverse 5′- CAGCGGCCCATGTAACCGAGCTGAGTGCTGAG -3′

SIDT2 extracellular CRAC domain:

forward 5′- CACCCGTCAATACCACTGGCCAGCTCCGAGTCAAC -3′

reverse 5′- GTTGACTCGGAGCTGGCCAGTGGTATTGACGGGTG -3′

These mutations were directed to replace the tyrosines (176, extracellular and 576 transmembranal) for alanines. All constructs were fully sequenced before transfection. Underlined are the mutated codons.

### Cell culture

Human embryonic kidney (HEK293T) cells were purchased from the American Type Culture Collection (ATCC, Manassas, VA). Cells were grown on 35 mm culture dishes using DMEM(Invitrogen, Carlsbad, CA) supplemented with 10% of heat inactivated Fetal Bovine Serum (Wisent, premium quality, Canada), Penicillin-Streptomycin (Life technologies) and glutamine (Life technologies) in an incubator with humidity control at 37 °C and 5% of CO2. 24 hours before transfection the cells were incubated with trypsin and grown on circular coverslips then the cells were transfected by CaCl_2_ protocol with the plasmids for SIDT1-GFP or SIDT2-GFP as following. Using a mixture of 1 µg of DNA and 6 µl CaCl_2_ reaching 60 µl of final volume with distilled H_2_O next the mixture was added by dropping to a HeBS buffer (50 mM HEPES, 280 mM NaCl, 1.5 mM Na2HPO4, pH 7.05) and the final mixture was incubated for 30 minutes prior to replacing it withDMEM. The cells were incubated overnight with the transfection mixture and then replaced with fresh medium to perform the assays.

### Confocal microscopy and FRET assay

The confocal images were acquired using an Olympus Fv10i confocal microscope using the corresponding filter for each fluorophore (GFP, FM-464, DHE). For the colocalization analysis, we use specific dyes for each organelle, ER tracker (Invitrogen) for endoplasmic reticulum, Lysotracker for lysosomes, immunostaining using an antibody against golgin97 for Golgi apparatus, and FM-464 for plasma membrane. The FRET efficiency was determined by the sensitized emission method^44, 45^, using the confocal microscope we acquired images with the different emission filters. Cells incubated with dehydroergosterol DHE (10 µM) during 6 hours with humidity control at 37 °C and 5% of CO2 overexpressing SIDT1-GFP, SIDT-2GFP or control cells. The automatic FRET calculation was done by the microscope ASW 2.1 software. Images for TIRF measurement were acquired with an Olympus microscope with the TIRF module using blue laser (405 nm) for GFP excitation. The images were analyzed using ImageJ software and co-localization plugin to obtain the Pearson’s colocalization coefficients.

### Depletion of cholesterol in the plasma membrane

Overexpressing SIDT-GFP cells were incubated in Optimem with 1% of MβCD (w/v) for 1 hour for the depletion of cholesterol in the plasma membrane of the cells. After the incubation, the cells were rinsed three times with Krebs-Ringer solution and were maintained in this solution for the duration of the experiments.

### [^3^H]-Cholesterol Uptake Experiments

A standard procedure was employed to measure influx of [^3^H]-cholesterol^31^. Hek293 cells were plated in 35 mm Petri dishes and used at 95% confluence or higher (1.2 × 10^6^ cells). Cells were incubated with 10 mM methyl beta cyclodextrin (MβCD) to remove the cholesterol from plasma membrane. Cells were cultivated for 5 hours in serum-free media and exposed to 1 µC [^3^H]-cholesterol^31^. After 4-hour exposure, the medium was aspirated, and the cell sheet washed gently, five times, with serum-free media. At the end of the washout, the medium was aspirated, the cell sheet washed four times with PBS and harvested in 1.0 ml PBS. To assay total radioactivity 0.3 ml cell preparation was placed on a high-performance glass vial (Perkin Elmer) mixed with 1.0 ml Pico-Fluor solution (Perkin Elmer). All samples were counted in a Quantulus liquid scintillation spectrometer (Perkin Elmer). Counting rates were corrected for quenching with an external standard and are expressed in disintegrations per minute (dpm).

### Western Blot for membrane proteins

The membrane proteins were isolated using cell surface isolation kit (Pierce) in accordance with the manufacturer instructions using cells overexpressing SIDT1-GFP treated with MβCD and control cells (cells expressing SIDT1-GFP but not exposed to MβCD). Then the isolated membrane fraction proteins were analyzed by western blot, and the difference was determined by densitometry analysis using ImageJ software.

### Synthesis of the CRAC domains from SIDT1

The sequence containing the extracellular CRAC domain from SIDT1 was: (150)ASMAPLGAQYKLLVTKLKHFQLRTNVA(176). The sequence containing the transmembrane CRAC domain from SIDT1 was:(633)AIHVLASLALSTQIYYMGRFKIDLGI(659). We produced fusion proteins by attaching these sequences to the amino terminus of GFP by PCR. In red are highlighted the CRAC domains. The proteins were produced using the bac-to-bac baculovirus expression system (Invitrogen), purified before used in the microarray studies. Purification was attained using the Green Fluorescent Protein Chromatography Kit (Bio-Rad). Purity was confirmed by HPLC.

### Sterols microarrays

We utilized the total internal fluorescence microscopy (TIRFM) system from TIRFLabs in microarray studies^46^. Cholesterol, cholic acid, dehydrocholic acid, β-estradiol and testosterone were purchased from Sigma (St. Louis Missouri). The anti-GFP antibody was purchased from Alamone. All sterols were dissolved in ethanol to a final concentration of 10 ng. The solution was mixed with 2% of polyacrylamide and spotted manually using a multibarrel pipette on the glass of standard 1 mm thick microarray glass slides. The slide was placed on the closed perfusion chamber of the lg-TIRFM system^46^. Purified fusion proteins containing the extracellular and transmembrane CRAC domains from SIDT1 fused to GFP were used at a final concentration of 100 nM and perfused into the chamber. The reaction was allowed to take place for 5 minutes, and then the chamber was rinsed with at least 5 times its volume to remove unbound fusion proteins. As a positive control, some spots in the microarray were printed with anti-GFP antibody suspended in PBS solution and mixed with 2% polyacrylamide.

### Data and materials availability

All non-commercial plasmids described in this study are available through an MTA.

## Electronic supplementary material

Supplementary figures
