# Supplementary material for: A novel family of mammalian transmembrane proteins involved in cholesterol transport
Source: Sci Rep. 2017 Aug 7;7:7450. doi: 10.1038/s41598-017-07077-z (PMC5547113; doi:10.1038/s41598-017-07077-z)
Supplement: Supplementary file 1 — Supplementary figures [file 41598_2017_7077_MOESM1_ESM.pdf]

A novel family of mammalian transmembrane proteins involved in cholesterol transport.

Authors: Kevin M. Méndez-Acevedo[1], Julián Valdes[2], Alexander Asanov[3], Luis Vaca[1*]

Supplementary Materials

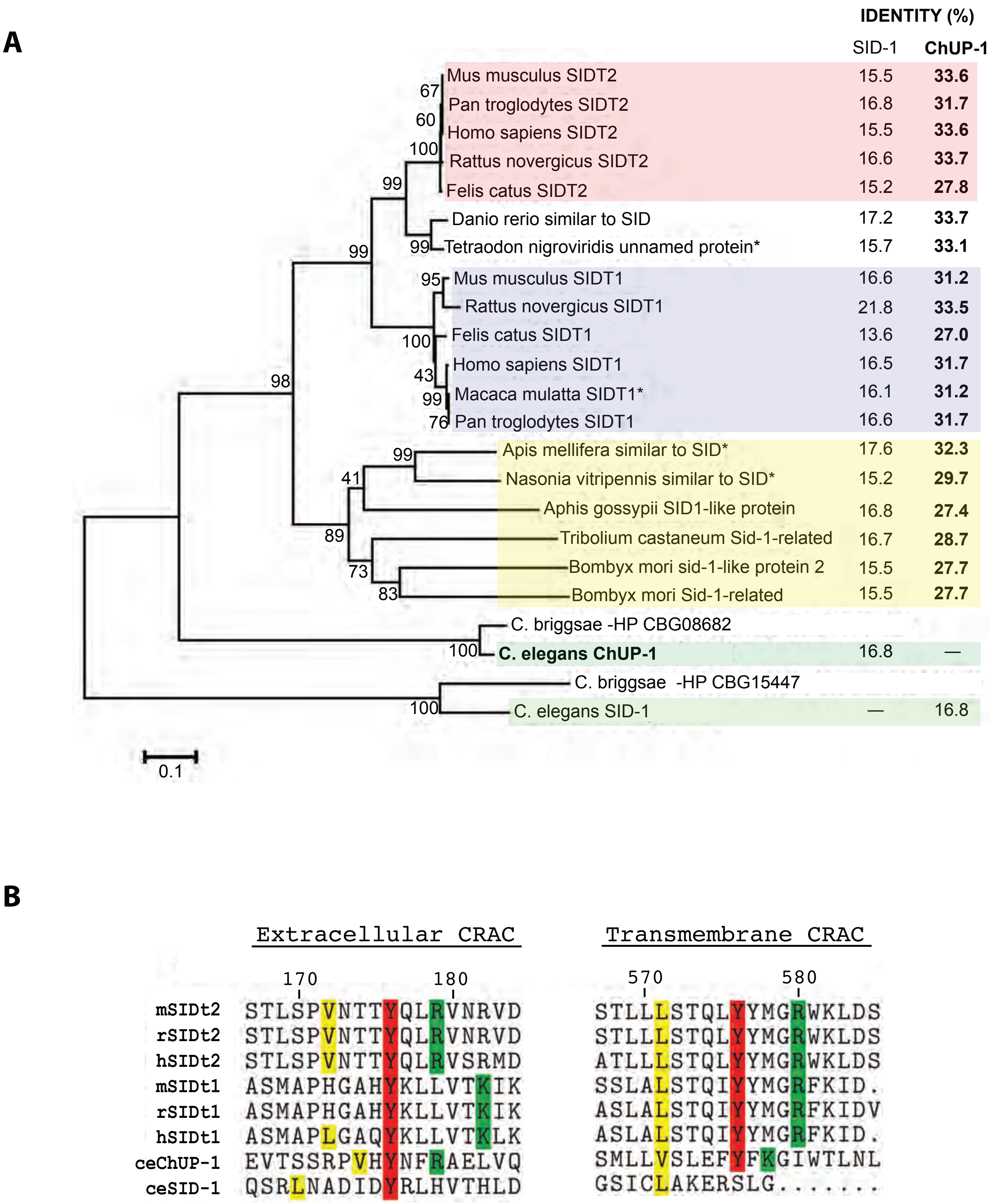

Supp. figure 1

A

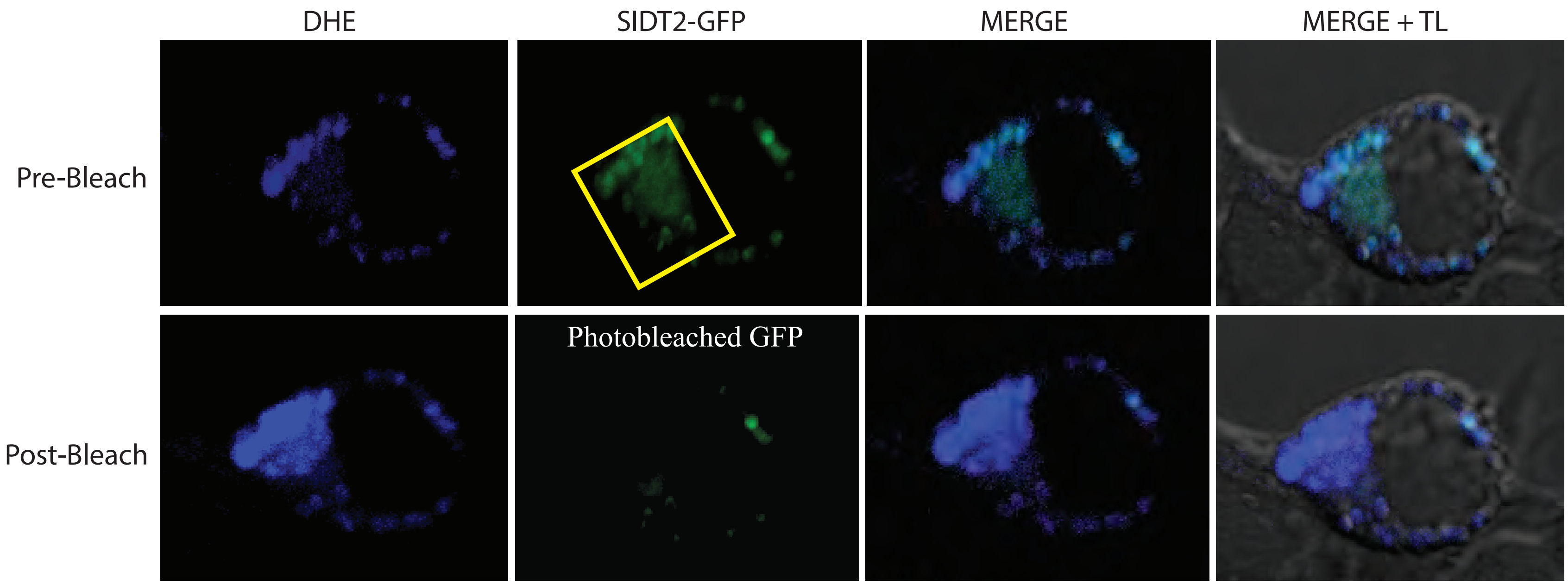

B

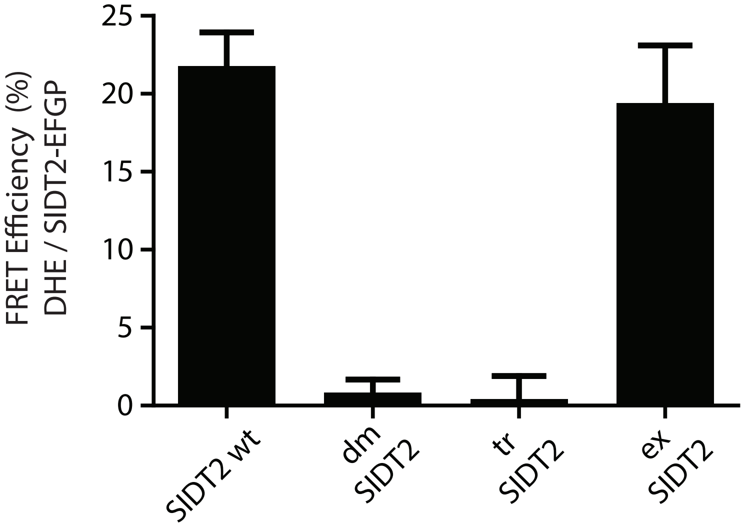

Supp. figure 2

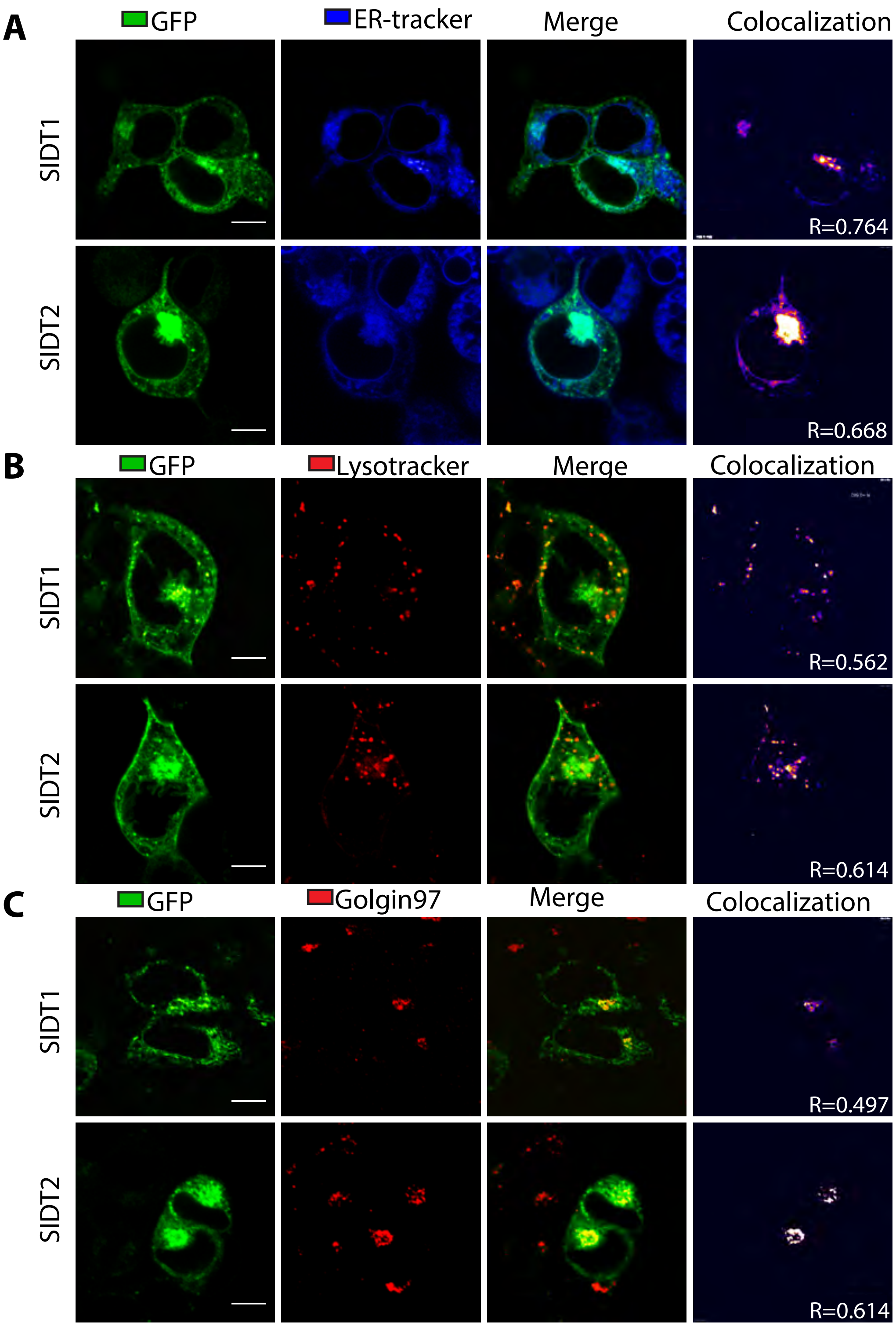

Supp. figure 3

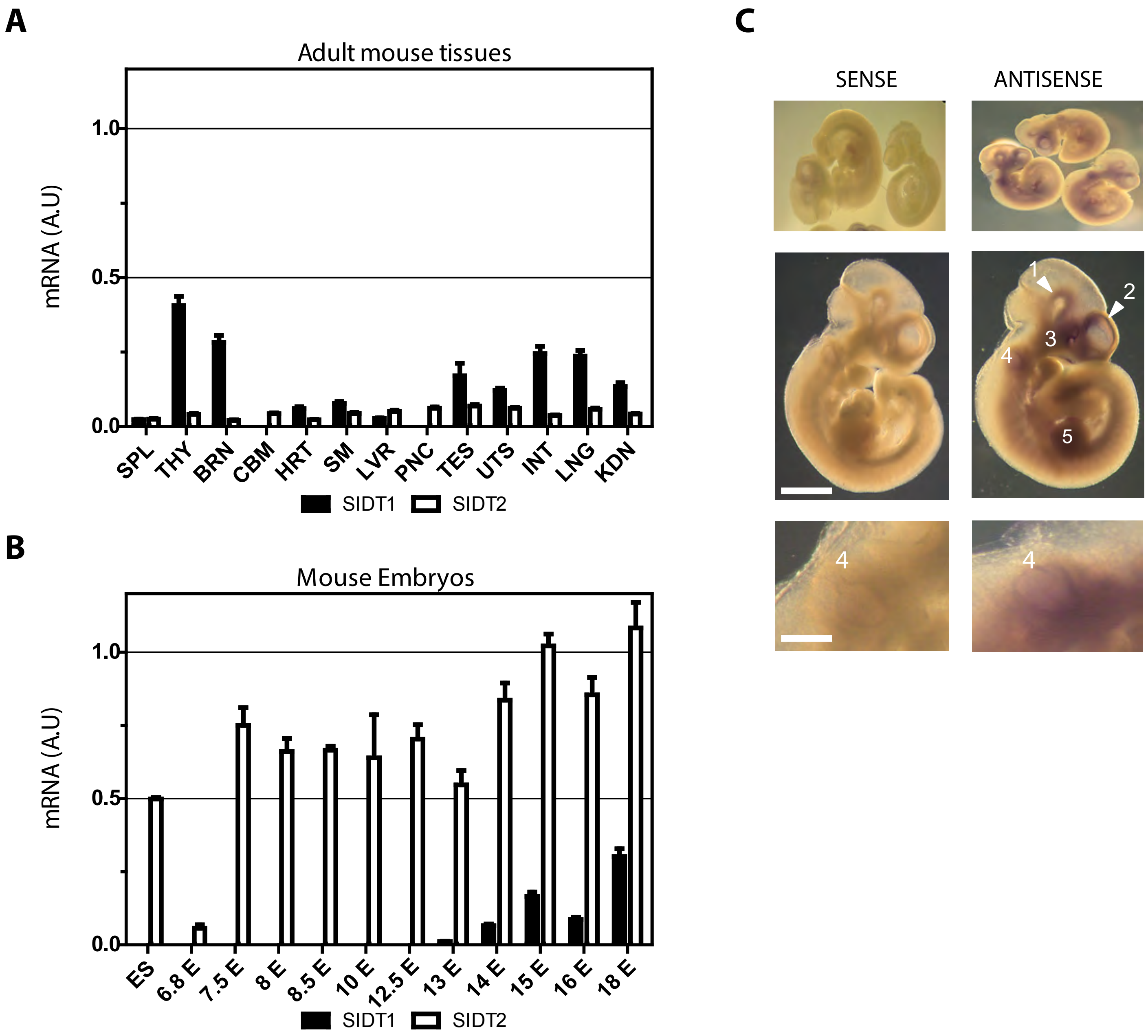

Supp. figure 4

Fig S1. Phylogenetic analysis of SIDT1 and SIDT2 genes. Protein sequences of *C. elegans* ChUP-1 homologues were aligned by ClustalW (MEGA5 (50)). **(A)** The multiple sequence alignment was then used to generate a phylogenetic tree by a maximum likelihood method using a WAG substitution model. Percentage of amino acid identity *vs C. elegans* SID-1 and ChUP-1 are presented on the right. Numbers in branches indicate bootstrap values. **(B)** Alignment of the conserved extracellular and transmembrane CRAC motifs (L/V-$X_{(1\text{–}5)}$-Y-$X_{(1\text{–}5)}$-R/K) in ChUP-1 homologue proteins. Numbers indicate amino acid position in CUP-1. m: *Mus musculus*; r: *Rattus norvegicus*; h: *Homo sapiens*; ce: *Caenorhabditis elegans*.

Fig S2. Intracellular FRET between SIDT2-GFP and DHE. (**A**), representative Förster energy transfer (FRET) experiments between SIDT2-GFP and dehydroergosterol (DHE) using acceptor photo-bleaching methods. Upper panels show the pre-bleach fluorescence and lower panels the post-bleach fluorescence. The yellow square indicates the area photo bleached. Notice that after photobleaching GFP the fluorescence of DHE increased only inside the area selected. The last panels to the right show the merge of fluorescence and transmitted light (TL). **(B)** Percentage of FRET efficiency obtained after photobleaching GFP. SIDT2 wild type (wt) is compared with the SIDT2 mutated at the tyrosine forming the transmembrane CRAC domain (tr-SIDT2) and the mutant on the extracellular CRAC domain (ex-SDIT2) as well as the double mutant (dm-SIDT2). Data shows the mean ± standard deviation of at least 25 independent measurements.

Fig S3. Co-localization of SIDT1 and SIDT2 with organelles. Confocal microscopy co-localization studies using cells expressing SIDT1-GFP or SIDT2-GFP. (**A**) Co-localization of SIDT1 and SIDT2 with ER-tracker (marker of the endoplasmic reticulum, ER). (**B**) Co-localization with lysotracker (marker of lysosomes). **(C)** Co-localization with golgin-97 (a protein resident of the trans Golgi network, TGN). R represents the Pearson's correlation coefficient.

Fig S4. Expression of SDIT1 and SIDT2 in mouse. Semiquantitative RT-PCR analysis to assess the expression levels of SIDT1 (black bars) and SIT2 (white bars) in **(A)** adult mouse tissues or in **(B)** mouse embryos (between 6,8 and 18-day postcoitus). Adult tissues: SPL: spleen, THY: thyme, BRN: brain, CMV: cerebellum, HRT: heart, SM: skeletal muscle, LVR: liver, PNC: pancreas, TES: testicles, UTS: uterus, INT: intestine, LNG: lungs, KDN: kidneys. Notice that SIDT1 is detected only after 14E. **(C)** Localization of SIDT2 in mouse embryo at 11.5 days postcoitus. hybridized with 3'UTR sense probe (control) and with 3'UTR antisense probe. 1:base plate, 2:telencephalon, 3:eye, 4:otic vesicle, 5:liver. Normalization against GAPDH levels in RT-PCR analysis. Scale bar: 1mm. All data shows mean ± standard deviation.
